# Significance of Heavy-Ion Beam Irradiation-Induced Avermectin B1a Production by Engineered* Streptomyces avermitilis*

**DOI:** 10.1155/2017/5373262

**Published:** 2017-01-24

**Authors:** Shu-Yang Wang, Yong-Heng Bo, Xiang Zhou, Ji-Hong Chen, Wen-Jian Li, Jian-Ping Liang, Guo-Qing Xiao, Yu-Chen Wang, Jing Liu, Wei Hu, Bo-Ling Jiang

**Affiliations:** ^1^Institute of Modern Physics, Chinese Academy of Sciences, 509 Nanchang Rd., Lanzhou, Gansu 730000, China; ^2^Lanzhou University, 222 South Tianshui Road, Lanzhou, Gansu 730000, China; ^3^Institute of Veterinary Drug Quality Inspection of Shandong Province, Jinan 250022, China

## Abstract

Heavy-ion irradiation technology has advantages over traditional methods of mutagenesis. Heavy-ion irradiation improves the mutation rate, broadens the mutation spectrum, and shortens the breeding cycle. However, few data are currently available regarding its effect on* Streptomyces avermitilis* morphology and productivity. In this study, the influence of heavy-ion irradiation on* S. avermitilis* when cultivated in approximately 10 L stirred-tank bioreactors was investigated. The specific productivity of the avermectin (AVM) B1a-producing mutant* S. avermitilis* 147-G_5_8 increased notably, from 3885 to 5446 *μ*g/mL, approximately 1.6-fold, compared to the original strain. The mycelial morphology of the mutant fermentation processes was microscopically examined. Additionally, protein and metabolite identification was performed by using SDS-PAGE, 2- and 3-dimensional electrophoresis (2DE and 3DE). The results showed that negative regulation gene deletion of mutants led to metabolic process upregulating expression of protein and improving the productivity of an avermectin B1a. The results showed that the heavy-ion beam irradiation dose that corresponded to optimal production was well over the standard dose, at approximately 80 Gy at 220 AMeV (depending on the strain). This study provides reliable data and a feasible method for increasing AVM productivity in industrial processes.

## 1. Introduction

Avermectins (AVMs) are metabolites from* Streptomyces avermitilis* [1]. This species' secondary metabolites have drawn great interest due to their unique structures and broad insecticide and anthelmintic activities [2–6]. The fermentation industry is attempting to improve the yield of AVM for future applications. Previous studies have shown that such products are positively affected by expression changes in housekeeping gene [7–10]. However, artificial manipulation of the genes involved in the AVM production does not readily lead to a mutant with satisfactory AVM B1a mass production. Furthermore, this genetic manipulation must be extremely precise, which would have high cost and potential for unsafe process operation. Some manipulations cannot be effectively scaled up for production in a fermentation plant. Although ^12^C^6+^ heavy-ion irradiation plays a key role in bioprocessing and biofermentation design and scale-up, no work has been done on heavy-ion irradiation and its effect on the growth and AVM productivity of* S. avermitilis.*

In this study, mutants with high productivity were used for process optimization with ^12^C^6+^ heavy-ion irradiation. The energy and dosage of ^12^C^6+^ heavy ions affected the phenotypes of survivors and generated mutants. Depending on the desired AVM or AVM B1a product, the original energy input and dose for a given bioprocess varied. Optimal key parameters, however, strongly correlated with the culture process and the operating parameters. To ensure the high production of the AVM B1a metabolite by mutants, it must be noted that the productivity of a bioprocess is not only determined by each specific process and its associated parameters, but also significantly impacted by the dry cell weight, kinetics, agitation speed, the initial fair values (*kLa*), dissolved oxygen (DO), and viscosity within the bioreactor [11–15]. The various process parameters and ingredients that influence the AVMs and AVM B1a production of the original strain have been extensively described in the literature [16–20]. Furthermore, many studies have shown that the DO, the oxygen supply, and the agitation speed are interrelated in varied culture conditions [21–23]; decoupling of these factors is often obtained by adjustment of the stirred-tank bioreactor stir speed, the type and number of stirred-tank bioreactors, the gas flow rate, cell growth, and the morphology of mycelia; metabolite biosynthesis variably depends on these factors [24–26]. Because the DO was found to be the primary factor affecting microbial metabolite production, most culture processes adopt a DO-control strategy by adjusting agitation. Future investigations should focus on identifying the essential regulating factors in the culture process [27, 28], which have vital importance for achieving high cultivation performance.

In the present work, heavy-ion irradiation and its effect on the growth and specific productivity of* S. avermitilis* were studied. The effects of the energy and dose of ^12^C^6+^ heavy ions were determined and then these parameters have been used to develop a customized bioreactor and bioprocess design. These findings may prove to be invaluable and helpful in industrial design and scale-up or for other applications.

## 2. Materials and Methods

### 2.1. Experimental Setup and Heavy-Ion Beam Irradiation

Heavy-ion beam experimental setups were employed as previously described [29]. The extraction time of the ^12^C^6+^ heavy ions (approximately 140 AMeV, 180 AMeV, and 220 AMeV of energy) was approximately 3 s, the priming dose was 80 Gy, and the dose rates were up to 10 Gy/min. In this study, the operating parameters were as follows: the radiation energy input was 140, 180, and 220 AMeV; the temperature of the ^12^C^6+^ heavy-ion beams was <35°C under these conditions [30, 31].

### 2.2. Strain and Culture Medium

The original* S. avermitilis* strain (AV-J-AO) was obtained from the Industrial Microbial Culture Collection Center of Gansu Province, China. The original culture medium consisted of the followings: KNO_3_ 1.5 g/L, K_2_HPO_4_·3H_2_O 0.5 g/L, NaCl 0.5 g/L, FeSO_4_ 0.01 g/L, corn starch 25 g/L, yeast extract 2.0 g/L, and soluble agar 20 g/L in distilled water at pH 7.3 ± 0.1. The original seeding medium for mutant strains consisted of the followings: corn starch 40 g/L, yeast extract 5 g/L, soy flour 3.5 g/L, and CoCl_2_·6H_2_O 0.02 g/L in distilled water at pH 7.5 ± 0.1. The original fermentation medium consisted of the following: MgSO_4_·7H_2_O 0.6 g/L, K_2_HPO_4_·3H_2_O 0.6 g/L, CoCl_2_·6H_2_O 0.02 g/L, CaCO_3_ 2.5 g/L, and KCl 5 g/L in distilled water at pH 7.5 ± 0.1. All media were autoclaved at 121°C for 20 min.

### 2.3. Experimental Protocol for Mutant Strains

The ^12^C^6+^ heavy-ion irradiated spore solution was spread on the original seeding of mutant strains medium and cultivated to form colonies. After incubation for 6 days at 30°C, many single colonies with various morphologies were observed. Each colony was counted and isolated. A few isolates were selected as inoculants for fermentation at 30°C for 12 days to examine the specific productivity of AVM B1a.

### 2.4. Bioreactor Configuration

The geometric parameters of the bioreactor are as follows: diameter (*T*), 220 mm; liquid height (*H*), 89 mm; bottom type, flat; filled volume (VL), 7 L; capacity (*V*), 10 L; impellers, shear. The main cultivation conditions were as follows: temperature, 28°C; air flow rate, 50 L/min; stirring speed, 180–200 rpm beginning 3 h after inoculation. The DO was controlled above 20% in all fermentation processes.

### 2.5. Detection of the Lethality and Mutation Rates

After irradiating the inoculated plates, the lethality and mutation rates were calculated using the following equations:

(1) Survival  rate=TU×100%Positive  mutation  rateRM/T=MT×100%Negative  mutation  rateRP/T=PT×100%,

where *U* is the total number of colonies of the sample without treatment; *T* is the total number of colonies after treatment with plasma; *M* is the number of colonies of the mutant strains that produce less AVM B1a than the original strain; and *P* is the number of colonies of mutants that produce more AVM B1a than the original strain.

### 2.6. Mycelial Soluble Protein Extraction and Analysis

Intracellular protein extracts were prepared for analysis by 2- and 3DE based on the method of Jun et al. [32]. Proteins were visualized using Coomassie brilliant blue staining as described by Neuhoff et al. [33]. Spots of interest were excised, and in-gel digestion with trypsin was performed as described by Jun et al. [32].

### 2.7. Measurement of Cell Growth, Residual Dextrin, and AVM and AVM B1a Production

Three milliliters of culture broth was taken for each time point and centrifuged at 3,500 rpm for 10 min. The pellet was dried to constant weight at 110°C to measure the dry cell weight (DCW). The total dextrin consumption was determined using dextrin assay kit according to the manufacturer's instructions (Rongsheng Biotech. Ltd., Shanghai). AVM and AVM B1a were analyzed by HPLC (LC10A; Shimadzu, Japan).

### 2.8. Measurement of Fair Value

The initial fair values (*k*_*L*_*a*) in bioreactors were prepared for analysis by the non-steady-state method [34] and were calculated by the following equation:

(2) $$\begin{array}{c} \frac{dc}{dt}=k_{L}a\left(\;C^{*}-C\;\right), \end{array}$$

where *dc*/*dt* represents the DO change over time, *C*^*∗*^ denotes the saturated DO concentration, and *C* denotes the DO at a specific time point. The *k*_*L*_*a* value (h^−1^) was calculated as the slope of ln⁡(*C*^*∗*^ − *C*) versus time.

### 2.9. Determination of Agitation Speed

Determination of the agitation speed was calculated by the following equation (3):

(3) $$\begin{array}{c} V=\frac{\pi \times d\times N}{\text{1,000}}, \end{array}$$

where *d* is the turbine diameter (0.055 m here) and *N* is the agitation speed (runs per second).

### 2.10. Calculation of Dry Cell Weight (DCW)

The DCW was calculated with the following equations (4):

(4) DCW=∫t1t2DCW·dtDCWt2=DCWt1+DCWt1+DCWt22·t2−t1,

where DCW_*t*_1__ is the first point during the fastest logarithmic growth period and DCW_*t*_2__ is the last point. *t*_1_ and *t*_2_ are similarly described.

### 2.11. Determination of Oxygen Uptake Rate (OUR) and Specific Oxygen Uptake Rate (SOUR)

The oxygen consumption rate was determined as described by Garcia-Ochoa and Gomez [27]. The SOUR was calculated as the OUR was divided by the DCW at each time point.

### 2.12. Determination of Broth Viscosity and Cell Morphology

Broth viscosity was detected as described by Kato et al. [35]. At each sampling point, the morphology of samples placed on a slide was observed with a Nikon microscope (Nikon Eclipse TE2000-U, Tokyo, Japan).

### 2.13. Examination of Mutant Genetic Stability

For the first subculture, the mutant strains were streaked or spread and cultivated on medium for 6 days. For the second subculture, several single colonies were selected and streaked onto new solid medium plates for second 6-day cultivation. The same procedure was repeated for a total of six subcultures. After each subculture, the specific productivity of AVMs and AVM B1a was evaluated by fermentation.

### 2.14. Statistical Analysis

All data represent the result of three independent samples (flasks). The error bars indicate the standard deviation (SD) from the mean of experiments performed in triplicate. The data were analyzed by one-way analysis of variance followed by the Student-Newman-Keuls test. A two-tailed *p* value < 0.05 was considered statistically significant. SPSS 18.0 software for Windows was used for the statistical analyses (SPSS Inc. Chicago, IL, USA).

## 3. Results

### 3.1. Independent ^12^C^6+^ Heavy-Ion Irradiation Experiments

The ideal working protocol for the treatment of* S. avermitilis* spores by exposure to ^12^C^6+^ heavy-ion irradiation was explored. Based on the strain characteristics, the effect of heavy-ion beam irradiation on survival was evaluated for independent irradiation experiments. A number of post irradiation survivors after irradiation used a ^12^C^6+^ heavy-ion beam with an energy input of 140 AMeV at dose of 80 Gy at a dose rate of 10 Gy/min. The survival rate of the spores treated with 80 Gy was 13.7%; a very limited survival (8.4%) was obtained with 80 Gy of 180 AMeV ^12^C^6+^ heavy-ion irradiation. For further confirmation of these results, we increased the energy input to 220 AMeV at a dose of 80 Gy. There was only 3.4% survival under these conditions. Collectively, the data of survival rate for spores exposed to carbon ion beams of different energies are questionable. With increasing the energy of carbon ion beam, the value of linear energy transfer (LET) decreases. So the survival rate of spores should increase when increasing the beam energy. However, it is interesting that the data presented in our study showed an opposite tendency. Obviously, a lower energy of carbon ion beam is credited with a higher the value of LET, but the beam range is very short. It is for this reason that ^12^C^6+^ heavy-ion irradiation is the dose of radiation hot spot and cold spot effect, namely, some of the* S. avermitilis* spores were not irradiated or are exposed to very small doses of radiation.

### 3.2. Strain Morphology before and after ^12^C^6+^ Heavy-Ion Treatment at Different Energy Inputs and Dosages

After exposure to a high dose of high-energy ^12^C^6+^ heavy ions, the survivors may represent mutants. The survival rates of 13.7%, 8.4%, and 3.4% were determined after cultivation on medium for 6 days at 28°C. In total, there were more than 1000 single colonies growing on 100 plates. These colonies were clearly different from those that developed from nonirradiated spores. The survivors displayed multiple colony texture and colors, such as the flat straw, grey steamed bread, and white bald and taupe crimple forms. The colonies were cultivated from survivors of ^12^C^6+^ heavy-ion irradiation with energy inputs of 140, 180, and 220 AMeV at doses of 80 Gy, respectively. These sample points were randomly selected based on the survival rates before any data processing tasks were undertaken to avoid bias from human operators. The colonies from each of the 140-, 180-, and 220-AMeV treatments were classified into 8 groups, namely, AO-M_3_1 to AO-M_3_8, HS-P_1_1 to HS-P_1_8, and 147-G_5_1 to 147-G_5_8, respectively; the corresponding data are shown in Tables 1, 2, and 3, respectively.

### 3.3. Specific Production of AVMs and AVM B1a

The AVM and AVM B1a specific productivity of the nonirradiated original strains and the 24 selected mutant strains was determined by fermentation experiments in approximately 1 L stirred-tank bioreactors. For the nonirradiated original strain* S. avermitilis*, the yield values of AVM B1a and total AVMs, *Y*_AVM B1a_, and *Y*_Total AVMs_ were 3885 ± 180 and 4530 ± 270 *μ*g/mL, respectively, based on 3 replicates.  Table 1 shows the specific AVM productivity of strains irradiated with ^12^C^6+^ heavy-ion beam with an energy input of 140 AMeV at a dose of 80 Gy as a percentage of the original strain productivity. Among the randomly selected and examined strains, AO-M_3_1 showed the highest *Y*_AVM B1a_, which was over 1.48-fold higher than that of the nonirradiated original strain. Moreover, the *Y*_Total AVMs_ of AO-M_3_1 also increased by approximately 1.24-fold compared to that of nonirradiated original strain. Two other mutant strains, AO-M_3_2 and AO-M_3_8, showed 1.03-fold and 1.06-fold higher AVM specific productivity than the original strain, respectively.  Table 2 shows the AVM specific productivity of the strains that were irradiated with ^12^C^6+^ heavy-ion beam at an energy input of 180 AMeV and a dose of 80 Gy relative to the original strain. Among the randomly selected and examined strains, HS-P_1_8 showed the highest *Y*_AVM B1a_, which was over 1.44-fold higher than that of the original strain.  Table 3 shows the AVM specific productivity of the strains irradiated with ^12^C^6+^ heavy ions at an energy input of 220 AMeV and a dose of 80 Gy relative to the original strain. Among the randomly selected and examined strains, 147-G_5_8 shows the highest *Y*_AVM B1a_, which was over 1.76-fold higher than that of the original strain.

### 3.4. The Mutation Rate and Positive Mutation Rate

Tables 1, 2, and 3 show the ratios of *Y*_AVM B1a_ to *Y*_Total AVM_ (**γ**) for all of the mutants and for original strain* S. avermitilis*. The value of a metabolite production for the original strain was 22%, 25%, and 41%; for most mutants, it changed by more than 15%. The specific productivity of the* S. avermitilis* mutants in this determination had approximately 5% random error, based on the average data from quintuplicate measurements. Thus, only strains with greater than 5% increases or decreases in *Y*_AVM B1a_ or *Y*_Total AVMs_ compared to the original strain were identified as “*Positive*” or “*Negative*” mutants, respectively. Based on the specific productivity of 24 strains and the colony number of each group, the positive mutation rate *R*_*M*/*T*_ was estimated as 18.1%, 24.6%, and 22.8% for the 140-, 180-, and 220-AMeV treatments, respectively. The negative mutation rate *R*_*P*/*T*_ was estimated to be 4.9%, 12.7%, and 12.3% for the 140-, 180-, and 220-AMeV treatments, respectively, as shown in Tables 1, 2, and 3, respectively.

### 3.5. Kinetics of Original Strain and AO-M_3_1, HS-P_1_8, and 147-G_5_8 Growth in Shake Flasks

**Figure 1:**
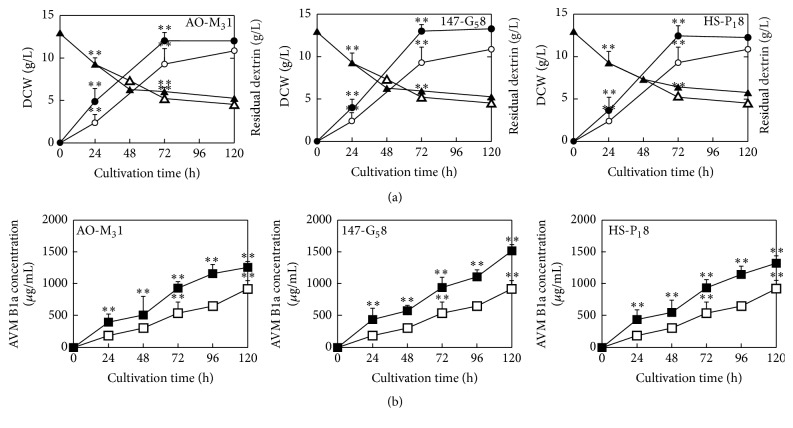
Growth kinetics of the original strain and the mutant strains AO-M_3_1, HS-P_1_8, and 147-G_5_8 cultivated with shaking. (a) Time course of the cell dry weight and dextrin consumption of the original strain and the mutant strains AO-M_3_1, HS-P_1_8, and 147-G_5_8. Left panel, comparison of the AO-M_3_1 mutant (black circles and black triangles) with original strain* S. avermitilis* (white circles and white triangles). Middle panel, comparison of the 147-G_5_8 mutant (black circles and black triangles) with original strain* S. avermitilis* (white circles and white triangles). Right panel, comparison of the HS-P_1_8 mutant (black circles and black triangles) with original strain* S. avermitilis* (white circles and white triangles). (b) Time profiles of the AVM B1a specific productivity of original strain and AO-M_3_1, HS-P_1_8, and 147-G_5_8 mutant. On the left is comparison of AO-M_3_1 mutant (black box) with the original strain* S. avermitilis* (white box), the comparison of 147-G_5_8 mutant (black box) with the original strains* S. avermitilis* (white box) is in the middle, and on the right is comparison of HS-P_1_8 mutant (black box) with the original strains* avermitilis* (white box). Data are the average of quintuplicate samples; the error bars indicate the standard deviations. All results were analyzed with Tukey's test (duplicate determinations, ^*∗∗*^*p* < 0.01).

Figure 1 shows the kinetic growth profiles of the AO-M_3_1, HS-P_1_8, and 147-G_5_8 mutant strains and the original strain. The biomasses of the AO-M_3_1, HS-P_1_8, and 147-G_5_8 mutants and the original strain began to increase after inoculation, reached maxima at 72 h, and then reached platform stage of growth. As shown in Figure 1(a), the growth of the AO-M_3_1, HS-P_1_8, and 147-G_5_8 mutants was much faster than that of the original type. The 147-G_5_8 mutant's maximum DCW was 13.7 ± 1.5 g/L, which was significantly higher than the 12.7 ± 0.7 g/L of the original strain. Dextrin consumption was in accord with each strain's cell growth; the sugar was consumed over 69.2% after 120 h by these mutants. This phenomenon may be explained as the higher biomass increase of mutants after more than 120 h compared to the original strain. As shown in Figure 1(b), the AVM B1a concentration continued to increase throughout the culture process of the mutants, while almost no AVM B1a was detected for the original strain. The maximal AVM B1a production by the 147-G_5_8 mutant was 1575 ± 120 *μ*g/mL at 120 h in convention shake flask culture. The results demonstrate the feasibility of AO-M_3_1, HS-P_1_8, and 147-G_5_8 mutant cultivation for producing a certain amount of AVM B1a, and the 147-G_5_8 mutant showed the best specific productivity.

### 3.6. Effects of Shaking Speed and Filling Volume on AO-M_3_1, HS-P_1_8, and 147-G_5_8 Mutant Growth in Flasks

**Figure 2:**
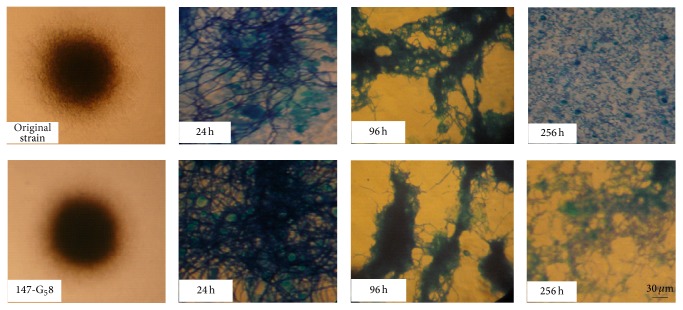
Comparison of the mycelial morphology between original strain* avermitilis* and the 147-G_5_8 mutant with optimized agitation at 250 rpm at different time points.

AVM B1a production was tested at different shaking speeds and filling volumes in baffled shake flasks. Shaking speed was the most important factor affecting AVM B1a production. At 250 rpm, the AVM B1a production of the AO-M_3_1 mutant ranged from 3889 to 3979 *μ*g/mL with filling volumes ranging from 20 to 80 mL. For the HS-P_1_8 mutant, production ranged from 4178 to 4238 *μ*g/mL with filling volumes ranging from 10 to 90 mL, and for the 147-G_5_8 mutant they ranged from 4560 to 4770 *μ*g/mL with filling volumes ranging from 10 to 100 mL. At 200 rpm, the AVM B1a production was only 3889–3927 *μ*g/mL for the AO-M_3_1 mutant, 4178–4216 *μ*g/mL for HS-P_1_8, and 4560–4630 *μ*g/mL for 147-G_5_8. The productivity of three mutants increased about 38~70 *μ*g/mL when the shaking speed increased from 200 to 250 rpm, but it varied only marginally with different filling volumes at a given shaking speed. The production of AVM B1a and total AVMs by the AO-M_3_1, HS-P_1_8, and 147-G_5_8 mutants was greater than that by the original strain.  Figure 2 shows that the 147-G_5_8 cellular morphology became much more hairy, with many thick hyphae, in contrast to the original strain, in which the majority of hyphae formed large, dense, and uniform pellets. After 256 h of cultivation in shake flasks, the original strain exhibited the hyphal autolysis obvious phenomenon.

### 3.7. Identification of the Most Highly Expressed Soluble Proteins in the Original Strain, HS-P_1_8, and 147-G_5_8 Mutants

**Figure 3:**
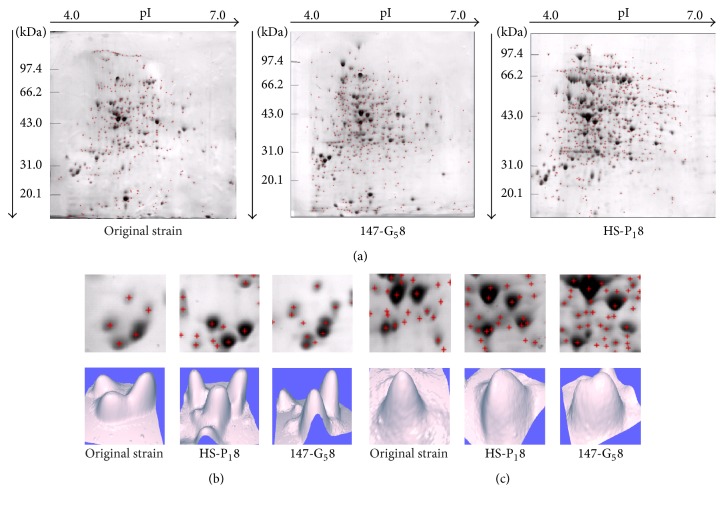
2DE and 3DE gels of soluble proteins to determine the most highly expressed proteins of the* S. avermitilis* original strain, HS-P_1_8, and 147-G_5_8 mutants. (a) The soluble proteins with the highest expression levels in original strain, HS-P_1_8, and 147-G_5_8 mutants were identified on 2DE gels. (b) The 3 strains were analyzed by 3DE in the ranges of 29 kDa and 4.2 pI. (c) The 3 strains were analyzed by 3DE in the ranges of 40.5 kDa and 5.1 pI. The figure shows that the expression of the 147-G_5_8 mutant was higher than that of original strain, although expression was only weakly observed.

A 2DE technique was used to identify the protein with the highest expression in the proteome of each mutant.  Figure 3(a) shows 2DE of the whole-protein fractions of the original strain, HS-P_1_8, and 147-G_5_8 mutants. The range of protein separation obtained by 2DE was 24–97 kDa and 4–7* *pI. 2DE of the original strain, HS-P_1_8, and 147-G_5_8 mutants yielded 345, 418, and 591 protein spots, respectively. All of these proteins were also presented in samples that were analyzed by PD-Quest. Comparing to original strain, the 147-G_5_8 had 3-fold higher concentrations with 29 147-G_5_8 protein spots. HS-P_1_8 had 29 protein spots with 2-fold higher concentrations than original strain. Among them, comparisons in the ranges of 29–40.5 kDa and 4.2–5.1 pI are notable differences.  Figure 3(b) shows that the 147-G_5_8 mutant had the highest overall expression and HS-P_1_8 the least, with weak overall expression observed.  Figure 3(c) also shows that the expression of the 147-G_5_8 mutant was higher than that of original strain, although expression was only weakly observed. These results demonstrate the feasibility of cultivating the 147-G_5_8 mutant to produce certain amount of AVMs and metabolites.

### 3.8. Impact of Agitation Speed on the Physiological Status of the AO-M_3_1, HS-P_1_8, and 147-G_5_8 Mutants When Cultivated in Stirred-Tank Bioreactors

**Figure 4:**
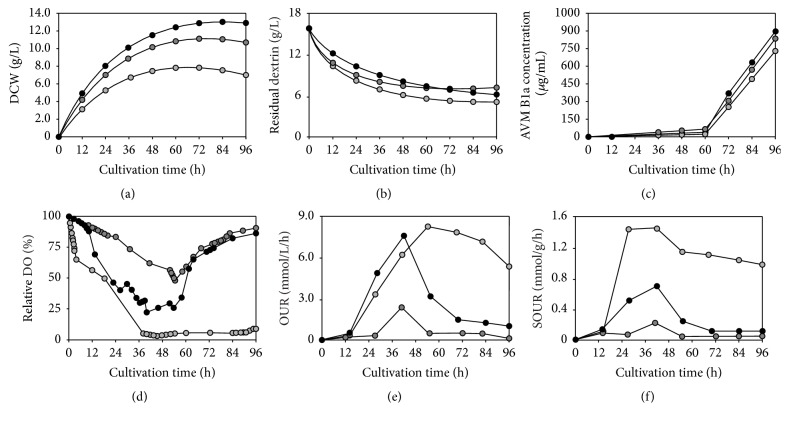
Time course of the physiological working capacity of 147-G_5_8 mutants cultivated in stirred-tank bioreactors with different agitation speeds. (a) Time course of cell dry weight with agitation speeds of 150, 250, and 350 rpm. (b) Time course of dextrin consumption with agitation speeds 150, 250, and 350 rpm. (c) Time course of the AVM B1a specific productivity with agitation speeds 150, 250, and 350 rpm. (d) Time course of the dash dotted line of DO-levels with agitation speeds 150, 250, and 350 rpm. (e) Time course of determination of oxygen uptake rate (OUR) with agitation speeds 150, 250, and 350 rpm. (f) Time course of determination of the specific oxygen uptake rate (SOUR) with agitation speeds 150, 250, and 350 rpm. Black circle symbol for 150 rpm speeds, grey circle symbol for 250 rpm speeds, and off-white circle symbol for 350 rpm speeds, respectively.

To investigate the effect of agitation intensity on the cultivation of the AO-M_3_1, HS-P_1_8, and 147-G_5_8 mutants in stirred-tank bioreactors, the cell growth, residual dextrin, AVM B1a yields, DO, OUR, SOUR, and broth viscosity at agitation speeds of 150, 250, and 350 rpm and at a fixed aeration rate of 1.0 vvm were examined in this study. As shown in Figure 4(a), the highest biomass production by the 147-G_5_8 mutant was 13.8 g/L at 150 rpm; this value slightly decreased at 250 rpm and dramatically dropped at 350 rpm, indicating a detrimental impact of a 350 rpm agitation speed on growth. This pattern was also observed for the AO-M_3_1 and HS-P_1_8 mutants (data not shown). As shown in Figure 4(b), dextrin consumption by the 147-G_5_8 mutant was similar at different agitation speeds and was completed at 96 h, after the biomass productivity reached its maximum. The pattern of consumption was similar for the AO-M_3_1 and HS-P_1_8 mutants (data not shown). As shown in Figure 4(c), AVM B1a production by the 147-G_5_8 mutant was higher at a relatively lower agitation speed; 896 *μ*g/mL was obtained at 150 rpm, 816 *μ*g/mL was obtained at 250 rpm, and 738 *μ*g/mL was obtained at 350 rpm, which was approximately 21.4% lower than what was achieved at 150 rpm. When the agitation speed was increased to 400 rpm, the production of AVM B1a by the 147-G_5_8 mutant decreased dramatically to 473 *μ*g/mL; this phenomenon was also exhibited by the AO-M_3_1 and HS-P_1_8 mutants (data not shown). It would be interesting to determine the detrimental effect of an agitation speed greater than 350 rpm on the cellular physiology and metabolism of the AO-M_3_1, HS-P_1_8, and 147-G_5_8 mutant strains. The 147-G58 mutant DO, OUR, and SOUR were recorded over time, as shown in Figures 4(d), 4(e), and 4(f), respectively. The dotted, dashed line representing DO remained above 25% throughout the culture process in stirred-tank bioreactors, guaranteeing a sufficient oxygen supply. The highest OUR and SOUR values, 7.3 mmol/L/h and 0.7 mmol/g/h, respectively, occurred at 42 h. Overall, a higher agitation speed resulted in lower OUR and SOUR values for the mutants, indicating weak respiration activity, which was in agreement with the rapid cell growth, lower biomass accumulation, and AVM B1a formation. The exception was an agitation speed of 350 rpm, at which the AO-M_3_1 (data not shown), HS-P_1_8 (data not shown), and 147-G_5_8 mutants started to rapidly synthesize AVM B1a and showed an abnormally high OUR. The broth viscosity was over time during culture of the 147-G_5_8 mutant in stirred-tank bioreactors. The viscosity reached its maximum at 72 h in all experimental conditions before decreasing, following a similar pattern as AVM B1a production. It should be noted that, at 350 rpm, the higher agitation speed caused hyphal fragmentation, which may correlate with higher broth viscosity.

### 3.9. The Initial Fair Values (*k*_*L*_*a*) Influence the Physiological Working Capacity Status of the AO-M_3_1, HS-P_1_8, and 147-G_5_8 Mutants

The initial fair values (*k*_*L*_*a*) of the AO-M31, HS-P18, and 147-G_5_8 mutants were determined. As shown in Table 4, the AO-M_3_1, HS-P_1_8, and 147-G_5_8 mutant cells showed rapid growth after inoculation and reached maximal values at 70 h, following the oxygen supply capacity of the cultivation system in all 3 cases. It would be interesting to determine the physiological working capacity status of the AO-M_3_1, HS-P_1_8, and 147-G_5_8 mutants, given that they seem to be only slightly influenced by oxygen supply. The available data show that the maximal biomass-specific productivity of mutants at 150 rpm and 1.10 vvm was slightly higher than at 150 rpm and 0.8 vvm, indicating that the higher initial fair values (*k*_*L*_*a*) reflect slightly increased biomass accumulation and dextrin consumption.

### 3.10. Determination of the Genetic Stability of Mutants

As shown in Table 5, the AO-M_3_1, HS-P_1_8, and 147-G_5_8 mutants were evaluated for their genetic stability in terms of AVM and AVM B1a specific productivity (under optimization fermentation parameters). The data show that these mutants could maintain their higher specific productivity after the sixth generation. Moreover, their specific productivity was sustained even as the growth rate increased. The sixth generation of 147-G_5_8 exhibited *Y*_AVM B1a_ and *Y*_Total AVMs_ values of 5446 ± 230 and 11089 ± 460 *μ*g/mL, respectively. These results suggest that the specific productivity of the strain increased in a dose-dependent manner at 220 AMeV of energy input with 80 Gy dose.

## 4. Discussion

In recent years, researchers have utilized traditional molecular biological mutation and selection strategies to obtain improved strains of* S. avermitilis *[36–39]. The new heavy-ion irradiation mutagenesis technique requires input from physics, chemistry, life science, and agronomy [40–42]. However, little data are currently available regarding the effect of heavy-ion irradiation on the production of AVMs and AVM B1a by* S. avermitilis*. Many of the details and effects of this mutagenesis technique were not resolved until now. This scientific report contains a proof-of-concept for the application of different energy levels and doses of ^12^C^6+^ heavy ions to generate* S. avermitilis* mutants with high AVM yields to achieve new breakthroughs in the theory and to apply key technology for the breeding and fermentation of these mutants in industrial applications.

The survival rate of* S. avermitilis* is closely related to the mutations that occur after exposure to different energies and doses of ^12^C^6+^ heavy ions. Generally speaking, a microbial survivor population of 15% or less may contain mutants. In this study, the original energy and dose of ^12^C^6+^ heavy ions were determined, and the resulting survival rates of 13.7%, 8.4%, and 3.4% were compared using a representative set of experimental data. The most prominent feature of most survival curves is a deviation from the dotted line; in other words, the energy- and dose-response curve typically shows shoulder [43–45]. Above an irradiation dose of 80 Gy with energy of 140 AMeV, the surviving fraction does not further increase but rather decreases. As expected, treatment with ^12^C^6+^ heavy ions affected survival; an increase in the irradiation energy or dose leads to a decrease in survival, with a final survival value of 3.4% at the strongest tested irradiation. Unexpectedly, as shown in Figures 1(a) and 4(a), we found that the 147-G_5_8 mutants reenter the cell cycle, which is defined as the time period required for cells to grow and divide, rather than completely losing their proliferation capacity [46, 47]. The specific productivity was determined by considering the DCW as the integrated biomass, thus producing integral of the DCW. The growth of the AO-M_3_1, HS-P_1_8, and 147-G_5_8 mutants was comparable. As shown in Figure 1, treatment with 220 AMeV and 80 Gy (147-G_5_8) led to 1.1-fold increase in DCW and 1.7-fold increase in the AVM B1a productivity of these mutants. Tables 1, 2, 3, and 5 show that the mutants within the bulk sample are genetically stable, showing AVMs production levels that increased to approximately 1.6 times that of the original strain. Despite the initial survival rates of 13.7%, 8.4%, and 3.4%, the growth of the AO-M_3_1, HS-P_1_8, and 147-G_5_8 mutant strains was not affected, and their production of AVM B1a and their DCW values both increased considerably.

The HS-P_1_8 and 147-G_5_8 mutants originate from the same parental strain, and their cultivation performance and handling characteristics are similar. We performed a comprehensive study of the protein expression levels over the growth curve, thus making it reasonable to compare how these mutants were affected by treatment with ^12^C^6+^ heavy ions. As shown in Figure 3, 2DE and 3DE were used to identify the most highly expressed soluble proteins in the HS-P_1_8 and 147-G_5_8 mutants. One large and distinct spot was presented in all three mutants, which revealed the existence of a potential highly expressed gene that may be a key gene in AVM and AVM B1a biosynthesis. Due to the DNA damage caused by treatment with ^12^C^6+^ heavy ions, DNA repair processes are activated. DNA repair is positively affected by housekeeping genes, such as* aveC*,* aveD*,* aveI*,* atrA*, and* aveR *which are not typically considered to be housekeeping genes; the “housekeeping” term is one typically reserved for genes whose function is needed for the growth and viability of an organism; these (particularly* aveC, I, and R*) are not essential and are more specifically associated with avermectin production [48–50]. Heavy-ion beam irradiation led to metabolic process upregulating expression of protein, decreasing synthesis inhibition of avermitilis B1a, and improving the productivity of avermectin B1a. Unfortunately, there are no detailed studies of these genes. However, many in vitro kinetics studies utilizing cultivation in shake flasks have demonstrated that these housekeeping genes have beneficial effects. For 147-G_5_8, the values for *Y*_AVM B1a_ and *Y*_Total AVMs_ were 5446 ± 190 and 11089 ± 230 *μ*g/mL, respectively. The *Y*_AVM B1a_ and *Y*_Total AVMs_ for 147-G_5_8 and HS-P_1_8 were also significantly higher than those of the original mutant AO-M_3_1.

To further enhance the AVM and AVM B1a specific productivity of the AO-M_3_1, HS-P_1_8, and 147-G_5_8 mutants and to achieve bioreactor scale-up, the key engineering parameters involved in the operating process were thoroughly evaluated. Three mutants were used in shake flask culture, a simple system that was suitable for substantiating our findings. Parameters such as shaking speed, the OUR, and the SOUR were determined at different filling volumes. As shown in Figures 1, 3, and 4, the AVM B1a specific productivity of the mutants was observed to be much more sensitive to the shaking speed than to filling volume. A shaking speed of only 250 rpm led to normal mycelial growth in the AO-M_3_1 and HS-P_1_8 mutants, while the 147-G_5_8 mutant became much more hairy, with many thicker hyphae. This result demonstrates the dependence on shaking speed and highlights the beneficial effect of an optimal shaking speed on AVM B1a production. Numerous studies have indicated that thicker mycelia may increase oxygen and nutrient transfer within the core zone, thus increasing metabolic efficiency [51–54]. Such an effect may coincide with our observation of the correlation between thicker mycelia and higher AVM B1a production at a shaking speed of 250 rpm. More interestingly, our results showed that oxygen transfer has a marginal impact on AO-M_3_1, HS-P_1_8, and 147-G_5_8 cultivation.

There are several other parameters that affect AVM B1a production in bioreactor scale-up, such as DO, OUR, SOUR, agitation speed, or the initial fair value (*k*_*L*_*a*). The exact parameters that are used usually depend on the method of measurement and the area of research. How ^12^C^6+^ heavy-ion treatment at different energies and doses affects production depends on the unknown adaptations of the AO-M_3_1, HS-P_1_8, and 147-G_5_8 mutant strains and their response to cultivation in stirred-tank bioreactors. As discussed above, to further study the effects of various parameters on AVM B1a production in mutant strains, experiments with agitation speeds of 150, 250, and 350 rpm with a fixed aeration rate at 0.8–1.0 vvm were conducted in stirred-tank bioreactors. As shown in Table 4 and Figure 4, the initial fair values (*k*_*L*_*a*) increased with agitation speed, indicating a positive effect on both OUR and SOUR transfer capacity. Similarly, a higher agitation speed resulted in decreased DCW gain but increased antibiotic production by* Streptomyces clavuligerus*,* Streptomyces hygroscopicus*,* Enterobacter cloacae*, and* Bacillus thuringiensis*. Interestingly, our data showed that AVM B1a production in stirred-tank bioreactors was severely reduced at 350 rpm, reflecting an abnormally high SOUR, while dextrin consumption remained similar to other conditions. Our findings also showed that a higher agitation speed would tear mycelia at the zone near the stirrer and thereby reduce DCW or strongly inhibit secondary metabolism [28, 55, 56]. Our results showed a positive correlation between AVM B1a production and the forms of mycelia permitted by lower agitation intensity. Similarly, the intact pellet morphology had a profound correlation with AVM B1a production; specifically, the fluffy pellets elongated, known as crossover morphology. This morphology was consistent among the mutants and affected the oxygen uptake, the specific oxygen uptake, or the nutrient limitation in the inner area, which may be relieved by these mutants' thicker mycelia or reduced physiologically active pellet size compared to large pellets [57, 58]. Future research should determine whether the mycelial form and prolonged germination time are directly connected during submerged growth in stirred-tank bioreactors.

In conclusion, when using heavy-ion irradiation technology, a new green technology, to generate mutants that produce AVMs and AVM B1a, it is crucial for industrial applications to distinguish between strains with high and low production. This study provides the means to characterize the mutants obtained through ^12^C^6+^ heavy-ion irradiation and to optimize bioreactor and bioprocess design; it also demonstrates the relationship between the mutants and higher productivity. ^12^C^6+^ heavy-ion irradiation is a fairly new parameter in process engineering. It was found to affect both mycelial morphology and specific productivity. ^12^C^6+^ heavy-ion irradiation may provide a reliable and highly active approach to increase the AVM and AVM B1a productivity of* S. avermitilis* in industrial processes. Because of the predictable behavior these mutants showed in response to the energy and dosage of ^12^C^6+^ heavy-ion irradiation, customized bioreactor and bioprocess designs for different process appear to be feasible. Because the rheology of the bioprocess and the downstream product processing steps are heavily dependent on strain production ability, carefully determined ^12^C^6+^ heavy-ion irradiation parameters may prove to be invaluable. The radiation parameters and process data obtained in this study will be helpful for further studies regarding the effective industrial applications and will establish a standard model for such projects or scale-up, thereby facilitating market acceptance and recognition.

## Figures and Tables

**Table 1:** Comparison of strain-specific productivity after ^12^C^6+^ heavy-ion irradiation with energy input of 140 AMeV at a dose of 80 Gy (*n* = 5).

Group	Number of colonies	Strain	*Y*_AVM B1a_^*∗*^	*Y*_Total AVMs_^*∗*^	**γ** (%)^*∗∗*^
		The original strain AV-J-AO	1.00 ± 0.11	1.00 ± 0.09	40.3
AO-M_3_1	8	AO-M_3_1-1	1.44 ± 0.25	1.24 ± 0.15	47.1
		AO-M_3_1-2	1.52 ± 0.13	1.27 ± 0.18	51.4
AO-M_3_2	13	AO-M_3_2-1	1.15 ± 0.22	1.12 ± 0.26	40.9
		AO-M_3_2-2	1.18 ± 0.17	0.94 ± 0.12	42.5
*P*	*21*				
AO-M_3_3	17	AO-M_3_3-1	0.78 ± 0.03	0.36 ± 0.09	58.9
		AO-M_3_3-2	0.42 ± 0.07	0.58 ± 0.06	26.7
AO-M_3_4	16	AO-M_3_4-1	0.36 ± 0.06	0.42 ± 0.08	53.7
		AO-M_3_4-2	0.57 ± 0.02	0.51 ± 0.06	51.4
AO-M_3_5	27	AO-M_3_5-1	0.43 ± 0.07	0.40 ± 0.03	49.8
		AO-M_3_5-2	0.37 ± 0.04	0.43 ± 0.07	53.3
AO-M_3_6	8	AO-M_3_6-1	0.94 ± 0.02	0.97 ± 0.04	48.9
		AO-M_3_6-2	0.47 ± 0.04	0.32 ± 0.09	45.7
AO-M_3_7	9	AO-M_3_7-1	0.27 ± 0.03	0.17 ± 0.02	31.5
		AO-M_3_7-2	0.17 ± 0.05	0.07 ± 0.06	—
*M*	77				
AO-M_3_8	328	AO-M_3_8-1	1.07 ± 0.03	1.16 ± 0.05	63.2
		AO-M_3_8-2	0.93 ± 0.03	0.87 ± 0.11	38.3
*T*	*426*				
*R*_*M*/*T*_ = *M*/*T* = *18.1%*			*R*_*P*/*T*_ = *P*/*T* = *4.9%*		

^*∗*^ *Y*_AVM B1a_ and *Y*_Total AVM_ represent the percentage content of specific productivity of all mutant's *Y*_AVMs B1a_ and *Y*_Total AVMs_ to those of the original strain *S. avermitilis*, respectively. ^*∗∗*^*γ* (%) indicates the ratio of *Y*_AVMs B1a_ to *Y*_Total AVMs_.

**Table 2:** Comparison of strain-specific productivity after ^12^C^6+^ heavy-ion irradiation with energy input of 180 AMeV at a dose of 80 Gy (*n* = 5).

Group	Number of colonies	Strain	*Y*_AVM B1a_^*∗*^	*Y*_Total AVMs_^*∗*^	*γ* (%)^*∗∗*^
		The original strain AV-J-AO	1.00 ± 0.11	1.00 ± 0.09	40.3
HS-P_1_1	31	HS-P_1_1-1	1.34 ± 0.35	1.32 ± 0.17	52.4
		HS-P_1_1-2	1.32 ± 0.18	1.19 ± 0.15	46.3
HS-P_1_2	23	HS-P_1_2-1	1.09 ± 0.22	0.97 ± 0.31	37.5
		HS-P_1_2-2	1.26 ± 0.22	1.12 ± 0.19	39.7
*P*	*54*				
HS-P_1_3	21	HS-P_1_3-1	0.26 ± 0.02	0.15 ± 0.07	25.9
		HS-P_1_3-2	0.38 ± 0.04	0.45 ± 0.03	37.1
HS-P_1_4	25	HS-P_1_4-1	0.16 ± 0.01	0.05 ± 0.02	—
		HS-P_1_4-2	0.27 ± 0.04	0.31 ± 0.03	27.3
HS-P_1_5	31	HS-P_1_5-1	0.56 ± 0.03	0.43 ± 0.05	35.6
		HS-P_1_5-2	0.42 ± 0.04	0.57 ± 0.12	49.3
HS-P_1_6	11	HS-P_1_6-1	1.13 ± 0.06	0.83 ± 0.09	39.8
		HS-P_1_6-2	0.12 ± 0.04	0.08 ± 0.09	—
HS-P_1_7	17	HS-P_1_7-1	0.34 ± 0.03	0.21 ± 0.04	39.1
		HS-P_1_7-2	0.59 ± 0.05	0.47 ± 0.06	43.8
*M*	105				
HS-P_1_8	267	HS-P_1_8-1	1.54 ± 0.09	0.97 ± 0.03	67.3
		HS-P_1_8-2	1.34 ± 0.11	1.05 ± 0.15	43.9
*T*	*426*				
*R*_*M*/*T*_ = *M*/*T* = *24.6%*			*R*_*P*/*T*_ = *P*/*T* = *12.7%*		

^*∗*^ *Y*_AVM B1a_ and *Y*_Total AVM_ represent the percentage content of specific productivity of all mutant's *Y*_AVMs B1a_ and *Y*_Total AVMs_ to those of the original strain *S. avermitilis*, respectively. ^*∗∗*^*γ* (%) indicates the ratio of *Y*_AVMs B1a_ to *Y*_Total AVMs_.

**Table 3:** Comparison of strain-specific productivity after ^12^C^6+^ heavy-ion irradiation with energy input of 220 AMeV at a dose of 80 Gy (*n* = 5).

Group	Number of colonies	Strain	*Y*_AVM B1a_^*∗*^	*Y*_Total AVMs_^*∗*^	*γ* (%)^*∗∗*^
		The original strain AV-J-AO	1.00 ± 0.11	1.00 ± 0.09	40.3
147-G_5_1	18	147-G_5_1-1	1.61 ± 0.13	1.21 ± 0.15	61.5
		147-G_5_1-2	1.44 ± 0.09	1.31 ± 0.18	42.9
147-G_5_2	23	147-G_5_2-1	1.71 ± 0.22	1.27 ± 0.26	62.4
		147-G_5_2-2	1.43 ± 0.13	1.12 ± 0.07	63.7
*P*	*41*				
147-G_5_3	13	147-G_5_3-1	1.14 ± 0.03	0.78 ± 0.09	57.3
		147-G_5_3-2	1.32 ± 0.06	0.69 ± 0.11	48.8
147-G_5_4	18	147-G_5_4-1	1.25 ± 0.05	0.52 ± 0.07	50.1
		147-G_5_4-2	1.07 ± 0.02	0.61 ± 0.06	49.3
147-G_5_5	21	147-G_5_5-1	0.38 ± 0.03	0.07 ± 0.03	—
		147-G_5_5-2	0.96 ± 0.07	0.72 ± 0.02	37.3
147-G_5_6	11	147-G_5_6-1	0.87 ± 0.02	0.97 ± 0.04	32.7
		147-G_5_6-2	1.07 ± 0.11	0.43 ± 0.13	48.9
147-G_5_7	13	147-G_5_7-1	0.45 ± 0.03	0.27 ± 0.02	43.4
		147-G_5_7-2	0.13 ± 0.02	0.05 ± 0.01	—
*M*	76				
147-G_5_8	217	147-G_5_8-1	1.87 ± 0.03	1.26 ± 0.05	69.2
		147-G_5_8-2	1.65 ± 0.09	1.42 ± 0.12	61.4
*T*	*334*				
*R*_*M*/*T*_ = *M*/*T* = *22.8%*			*R*_*P*/*T*_ = *P*/*T* = *12.3%*		

^*∗*^ *Y*_AVM B1a_ and *Y*_Total AVM_ represent the percentage content of specific productivity of all mutant's *Y*_AVMs B1a_ and *Y*_Total AVMs_ to those of the original strain *S. avermitilis*, respectively. ^*∗∗*^*γ* (%) indicates the ratio of *Y*_AVMs B1a_ to *Y*_Total AVMs_.

**Table 4:** The influence of initial fair values (*k*_*L*_*a*), stir speed, and AVM and AVM_B1a_ production on the working capacity of the AO-M_3_1, HS-P_1_8, and 147-G_5_8 mutants under various experimental conditions.

Mutant	Agitation speed (rpm)	Aeration rate (vvm)	Initial fair value *k*_*L*_*a* values (h^−1^)	Tip speed (ms^−1^)	AVM_B1a_ production (*μ*g/mL)	Total AVMs production (*μ*g/mL)
AO-M_3_1	150	1.10	34.4	1.10	3979 ± 110	10366 ± 220
	150	0.80	39.8	1.10	3889 ± 120	9022 ± 120
	250	1.10	42.3	1.20	2254 ± 170	4563 ± 120
	350	1.10	52.6	1.40	1835 ± 120	3843 ± 110

HS-P_1_8	150	1.10	34.4	1.10	4078 ± 110	9762 ± 180
	150	0.80	39.8	1.10	4232 ± 130	9674 ± 210
	250	1.10	42.3	1.20	2164 ± 110	4713 ± 170
	350	1.10	52.6	1.40	1978 ± 100	3689 ± 120

147-G_5_8	150	1.10	34.4	1.10	4560 ± 110	10767 ± 230
	150	0.80	39.8	1.10	4770 ± 150	10936 ± 220
	250	1.10	42.3	1.20	2343 ± 130	4892 ± 170
	350	1.10	52.6	1.40	2183 ± 110	4685 ± 160

**Table 5:** Values of *Y*_AVM B1a_ and *Y*_Total AVMS_ for AO-M_3_1, HS-P_1_8, and 147-G_5_8 mutants for different subcultures (*n* = 5).

Mutant	Subcultures	2	3	4	6
AO-M_3_1	*Y*_AVM B1a_ (*μ*g/mL)	3948 ± 240	3721 ± 220	3820 ± 270	3750 ± 350
	*Y*_Total AVMS_ (*μ*g/mL)	10297 ± 290	10090 ± 330	10459 ± 210	10730 ± 280
HS-P_1_8	*Y*_AVM B1a_ (*μ*g/mL)	4750 ± 280	4657 ± 310	4759 ± 270	4665 ± 220
	*Y*_Total AVMS_ (*μ*g/mL)	10645 ± 380	9835 ± 260	11470 ± 330	10670 ± 290
147-G_5_8	*Y*_AVM B1a_ (*μ*g/mL)	5546 ± 240	5760 ± 290	5912 ± 210	5446 ± 230
	*Y*_Total AVMS_ (*μ*g/mL)	11320 ± 380	11982 ± 350	12067 ± 310	11089 ± 460

## References

[1] Burg R. W., Miller B. M., Baker E. E. (1979). Avermectins, new family of potent anthelmintic agents: producing organism and fermentation. *Antimicrobial Agents and Chemotherapy*.

[2] Bum Kim S., Goodfellow M. (2002). *Streptomyces avermitilis sp. nov.*, nom. rev., a taxonomic home for the avermectin-producing streptomycetes. *International Journal of Systematic and Evolutionary Microbiology*.

[3] Lee S. Y., Lee D. Y., Kim T. Y. (2005). Systems biotechnology for strain improvement. *Trends in Biotechnology*.

[4] Boxall A. B. A., Fogg L. A., Kay P., Blackwell P. A., Pemberton E. J., Croxford A. (2003). Prioritisation of veterinary medicines in the UK environment. *Toxicology Letters*.

[5] Boxall A. B. A., Kolpin D. W., Halling-Sørensen B., Tolls J. (2003). Are veterinary medicines causing environmental risks?. *Environmental Science and Technology*.

[6] Floate K. D., Wardhaugh K. G., Boxall A. B. A., Sherratt T. N. (2005). Fecal residues of veterinary parasiticides: nontarget effects in the pasture environment. *Annual Review of Entomology*.

[7] Witt D., Stackebrandt E. (1990). Unification of the genera *Streptoverticillium* and *Streptomyces*, and amendation of Streptomyces Waksman and Henrici 1943, 339(AL). *Systematic and Applied Microbiology*.

[8] Lin Y., Kieser H. M., Hopwood D. A., Chen C. W. (1993). The chromosomal DNA of *Streptomyces lividans* 66 is linear. *Molecular Microbiology*.

[9] Ikeda H., Ishikawa J., Hanamoto A. (2003). Complete genome sequence and comparative analysis of the industrial microorganism *Streptomyces avermitilis*. *Nature Biotechnology*.

[10] Wang W., Li X., Li Y., Li S., Fan K., Yang K. (2015). A genetic biosensor for identification of transcriptional repressors of target promoters. *Scientific Reports*.

[11] Yen H.-W., Hsiao H.-P. (2013). Effects of dissolved oxygen level on rapamycin production by pellet-form of *Streptomyces hygroscopicus*. *Journal of Bioscience and Bioengineering*.

[12] Mehmood N., Olmos E., Goergen J.-L. (2012). Decoupling of oxygen transfer and power dissipation for the study of the production of pristinamycins by Streptomyces pristinaespiralis in shaking flasks. *Biochemical Engineering Journal*.

[13] Gamboa-Suasnavart R. A., Valdez-Cruz N. A., Cordova-Dávalos L. E. (2011). The O-mannosylation and production of recombinant APA (45/47 KDa) protein from *Mycobacterium tuberculosis* in *Streptomyces lividans* is affected by culture conditions in shake flasks. *Microbial Cell Factories*.

[14] Xia X., Lin S., Xia X.-X., Cong F.-S., Zhong J.-J. (2014). Significance of agitation-induced shear stress on mycelium morphology and lavendamycin production by engineered Streptomyces flocculus. *Applied Microbiology and Biotechnology*.

[15] Bandaiphet C., Prasertsan P. (2006). Effect of aeration and agitation rates and scale-up on oxygen transfer coefficient, kLa in exopolysaccharide production from *Enterobacter cloacae* WD7. *Carbohydrate Polymers*.

[16] Fritz L. C., Wang C. C., Gorio A. (1979). Avermectin B1a irreversibly blocks postsynaptic potentials at the lobster neuromuscular junction by reducing muscle membrane resistance. *Proceedings of the National Academy of Sciences of the United States of America*.

[17] Zhang C., Albermann C., Fu X., Thorson J. S. (2006). The in vitro characterization of the iterative avermectin glycosyltransferase AveBI reveals reaction reversibility and sugar nucleotide flexibility. *Journal of the American Chemical Society*.

[18] Xu Z., Cen P. (1999). Stimulation of avermectin B1a biosynthesis in Streptomyces avermilitis by feeding glucose and propionate. *Biotechnology Letters*.

[19] Kitani S., Miyamoto K. T., Takamatsu S. (2011). Avenolide, a *Streptomyces* hormone controlling antibiotic production in *Streptomyces avermitilis*. *Proceedings of the National Academy of Sciences of the United States of America*.

[20] Awasthi A., Razzak M., Al-Kassas R., Harvey J., Garg S. (2012). An overview on chemical derivatization and stability aspects of selected avermectin derivatives. *Chemical and Pharmaceutical Bulletin*.

[21] Pfefferle C., Theobald U., Gürtler H., Fiedler H.-P. (2000). Improved secondary metabolite production in the genus Streptosporangium by optimization of the fermentation conditions. *Journal of Biotechnology*.

[22] Inoue K., Yoshimi Y., Hino T., Oka H. (2009). Simultaneous determination of avermectins in bovine tissues by LC-MS/MS. *Journal of Separation Science*.

[23] Ishida M., Haga R., Nishimura N., Matuzaki H., Nakano R. (1990). High cell density suspension culture of mammalian anchorage independent cells: oxygen transfer by gas sparging and defoaming with a hydrophobic net. *Cytotechnology*.

[24] Felipe M. S. (2005). Transcriptional profiles of the human pathogenic fungus *Paracoccidioides brasiliensis* in mycelium and yeast cells. *Journal of Biological Chemistry*.

[25] Abeles F. B., Morgan P. W., Saltveit M. E. (2012). *Ethylene in Plant Biology*.

[26] Gervais P., Molin P. (2003). The role of water in solid-state fermentation. *Biochemical Engineering Journal*.

[27] Garcia-Ochoa F., Gomez E. (2009). Bioreactor scale-up and oxygen transfer rate in microbial processes: an overview. *Biotechnology Advances*.

[28] Hortsch R., Stratmann A., Weuster-Botz D. (2010). New milliliter-scale stirred tank bioreactors for the cultivation of mycelium forming microorganisms. *Biotechnology and Bioengineering*.

[29] Lei Y., Zhao Y., Cheng R. (2013). Fluorescence emission from CsI(Tl) crystal induced by high-energy carbon ions. *Optical Materials*.

[30] Zhou X., Xie J.-R., Tao L. (2013). The effect of microdosimetric ^12^C^6+^ heavy ion irradiation and Mg^2+^ on canthaxanthin production in a novel strain of Dietzia natronolimnaea. *BMC Microbiology*.

[31] Wang S.-Y., Jiang B.-L., Zhou X. (2015). Study of a high-yield cellulase system created by heavy-ion irradiation-induced mutagenesis of Aspergillus Niger and mixed fermentation with Trichoderma reesei. *PLoS ONE*.

[32] Jun H., Kieselbach T., Jönsson L. J. (2012). Comparative proteome analysis of Saccharomyces cerevisiae: a global overview of in vivo targets of the yeast activator protein 1. *BMC Genomics*.

[33] Neuhoff V., Arold N., Taube D., Ehrhardt W. (1988). Improved staining of proteins in polyacrylamide gels including isoelectric focusing gels with clear background at nanogram sensitivity using Coomassie Brilliant Blue G-250 and R-250. *Electrophoresis*.

[34] Wang S.-J., Zhong J.-J. (1996). A novel centrifugal impeller bioreactor. II. Oxygen transfer and power consumption. *Biotechnology and Bioengineering*.

[35] Kato A., Kawzao S., Soh Y. (1978). Viscosity of the broth of tobacco cells in suspension culture: biomass production of tobacco cells (Part IV). *Journal of Fermentation Technology*.

[36] Zhang X., Chen Z., Li M., Wen Y., Song Y., Li J. (2006). Construction of ivermectin producer by domain swaps of avermectin polyketide synthase in S*treptomyces avermitilis*. *Applied Microbiology and Biotechnology*.

[37] Wang L.-Y., Huang Z.-L., Li G. (2010). Novel mutation breeding method for *Streptomyces avermitilis* using an atmospheric pressure glow discharge plasma. *Journal of Applied Microbiology*.

[38] Omura S., Ikeda H., Ishikawa J. (2001). Genome sequence of an industrial microorganism *Streptomyces avermitilis*: deducing the ability of producing secondary metabolites. *Proceedings of the National Academy of Sciences of the United States of America*.

[39] Jenke-Kodama H., Börner T., Dittmann E. (2006). Natural biocombinatorics in the polyketide synthase genes of the actinobacterium *Streptomyces avermitilis*. *PLoS Computational Biology*.

[40] Zhou X., Lu X.-H., Li X.-H. (2014). Radiation induces acid tolerance of *Clostridium tyrobutyricum* and enhances bioproduction of butyric acid through a metabolic switch. *Biotechnology for Biofuels*.

[41] Di C.-X., Han L., Zhang H. (2015). Diallyl disulfide attenuated carbon ion irradiation-induced apoptosis in mouse testis through changing the ratio of Tap73/Np73 via mitochondrial pathway. *Scientific Reports*.

[42] Jin X., Li F., Zheng X. (2015). Carbon ions induce autophagy effectively through stimulating the unfolded protein response and subsequent inhibiting Akt phosphorylation in tumor cells. *Scientific Reports*.

[43] Kanai T., Endo M., Minohara S. (1999). Biophysical characteristics of HIMAC clinical irradiation system for heavy-ion radiation therapy. *International Journal of Radiation Oncology Biology Physics*.

[44] Kramer M., Scholz M. (2000). Treatment planning for heavy-ion radiotherapy: calculation and optimization of biologically effective dose. *Physics in Medicine and Biology*.

[45] Tobias C. A., Blakely E. A., Alpen E. L. (1982). Molecular and cellular radiobiology of heavy ions. *International Journal of Radiation Oncology, Biology, Physics*.

[46] Goodhead D. T., Munson R. J., Thacker J., Cox R. (1980). Mutation and inactivation of cultured mammalian cells exposed to beams of accelerated heavy ions IV. Biophysical interpretation. *International Journal of Radiation Biology*.

[47] Pouget J.-P., Mather S. J. (2001). General aspects of the cellular response to low- and high-LET radiation. *European Journal of Nuclear Medicine*.

[48] Stutzman-Engwall K., Conlon S., Fedechko R. (2005). Semi-synthetic DNA shuffling of aveC leads to improved industrial scale production of doramectin by Streptomyces avermitilis. *Metabolic Engineering*.

[49] Gaisser S., Kellenberger L., Kaja A. L. (2003). Direct production of ivermectin-like drugs after domain exchange in the avermectin polyketide synthase of *Streptomyces avermitilis* ATCC31272. *Organic and Biomolecular Chemistry*.

[50] Hui T., Li Ping Z. (2011). Site-directed mutagenesis of *Streptomyces avermitilis* aveD gene. *Agricultural Science & Technology—Hunan*.

[51] Papagianni M. (2004). Fungal morphology and metabolite production in submerged mycelial processes. *Biotechnology Advances*.

[52] Brockman F. J., Kieft T. L., Fredrickson J. K. (1992). Microbiology of vadose zone paleosols in south-central Washington State. *Microbial Ecology*.

[53] Prosser J. I., Tough A. J. (1991). Growth mechanisms and growth kinetics of filamentous microorganisms. *Critical Reviews in Biotechnology*.

[54] Krull R., Wucherpfennig T., Esfandabadi M. E. (2013). Characterization and control of fungal morphology for improved production performance in biotechnology. *Journal of Biotechnology*.

[55] Bendig C., Weuster-Botz D. (2013). Reaction engineering analysis of cellulase production with *Trichoderma reesei* RUT-C30 with intermittent substrate supply. *Bioprocess and Biosystems Engineering*.

[56] Olmos E., Mehmood N., Husein L. H., Goergen J.-L., Fick M., Delaunay S. (2013). Effects of bioreactor hydrodynamics on the physiology of *Streptomyces*. *Bioprocess and Biosystems Engineering*.

[57] Galindo E., Larralde-Corona C. P., Brito T. (2005). Development of advanced image analysis techniques for the in situ characterization of multiphase dispersions occurring in bioreactors. *Journal of Biotechnology*.

[58] Jonsbu E., McIntyre M., Nielsen J. (2002). The influence of carbon sources and morphology on nystatin production by *Streptomyces noursei*. *Journal of Biotechnology*.

